# Herpes Simplex Virus Esophagitis in an Immunocompetent Patient: Navigating Diagnostic Challenges and Achieving Effective Treatment

**DOI:** 10.7759/cureus.109446

**Published:** 2026-05-22

**Authors:** Didi Mwengela, Nimah Ather, John Kunesh

**Affiliations:** 1 Department of Medicine, Division of Digestive Diseases, University of California, Los Angeles, Torrance, USA; 2 Torrance Memorial Department of Pathology and Clinical Laboratory, Torrance Memorial Medical Center, Torrance, USA

**Keywords:** acyclovir therapy, evaluations for dysphagia, herpes esophagitis, herpes esophagitis in immunocompetent, odynophagia

## Abstract

Herpes simplex virus (HSV) esophagitis is classically associated with immunocompromised states and is rare in immunocompetent patients, making the presentation of HSV esophagitis in an immunocompetent patient challenging to diagnose. We present the case of a 64-year-old immunocompetent male with a history of gastroesophageal reflux disease (GERD) who developed severe HSV esophagitis. The patient presented initially with severe retrosternal burning and inability to tolerate oral intake. Esophagogastroduodenoscopy revealed severe esophagitis, and biopsy confirmed herpes simplex virus infection. He was treated with acyclovir administered intravenously, followed by oral acyclovir therapy, along with proton pump inhibitor treatment, which resulted in rapid clinical improvement. This case highlights the importance of keeping viral etiologies on the differential for refractory esophageal symptoms, even in the absence of a history of immunocompromise, and underscores the role of endoscopy and biopsy in diagnostic evaluation. Early antiviral therapy was effective, demonstrating the need for prompt recognition and intervention for optimal outcomes. Increasing awareness of such cases can improve understanding and management of this uncommon condition.

## Introduction

Herpes simplex virus esophagitis, commonly seen in immunocompromised populations such as those with HIV/AIDS or undergoing chemotherapy, is rare in immunocompetent patients. Infectious esophagitis, once considered almost exclusively a disease of immunocompromised hosts, is increasingly recognized in immunocompetent individuals [1]. Patients typically present with odynophagia, retrosternal chest pain, heartburn, or fever [2,3]. In such cases, a definitive diagnosis is generally made via endoscopy with biopsy. Endoscopy often reveals multiple shallow ulcerations, and histopathology shows characteristic viral cytopathic changes [4-6]. Even in individuals without traditional risk factors, concurrent gastroesophageal reflux disease (GERD) and minor mucosal trauma may make immunocompetent individuals more susceptible to HSV esophagitis [2]. In this report, we present a 64-year-old man with a history of GERD who developed severe herpes esophagitis. This case demonstrates the diagnostic challenges and treatment approaches pertinent to managing HSV esophagitis in immunocompetent individuals. It demonstrates the need for increased clinical suspicion and prompt treatment to reduce complications and promote recovery.

## Case presentation

A 64-year-old male with a medical history notable for paroxysmal atrial fibrillation (on aspirin) and acid reflux presented with severe burning in his chest and inability to tolerate oral intake, including water, starting 2-3 days prior to presentation. The patient reported that the severity of acid reflux was unprecedented and noted a burning pain in the lower sternum associated with eating and drinking. He had previously visited the emergency room twice due to extreme pain. He was discharged on both occasions with oral proton pump inhibitor therapy without gastroenterology consultation. He returned to the emergency room as his severe symptoms continued despite the medication. He was not taking proton pump inhibitors prior to presentation. The patient reported consuming approximately one glass of wine daily and denied any history of tobacco use.

On physical exam, the patient appeared alert and oriented, in no acute distress. Vital signs were notable for a blood pressure of 151/63 mm Hg and a heart rate of 90 beats per minute. The respiratory rate was 20 breaths per minute with an oxygen saturation of 100% on room air. On examination, the lungs were clear to auscultation with non-labored respirations. The cardiac exam revealed a normal rate and regular rhythm without murmurs. The abdomen was soft, non-tender, and non-distended with normal bowel sounds, and there were no masses palpable. Neurologically, the patient was awake and oriented, with cranial nerves II-XII intact. Skin examination revealed warm, dry, and pink skin without rashes or lesions. Laboratory evaluation revealed a hemoglobin of 15.6 gm/dL, hematocrit of 44.6%, white blood cell count of 5.6 × 10³ cells/µL, and an HIV 1 and 2 screen was nonreactive.

**Figure 1:**
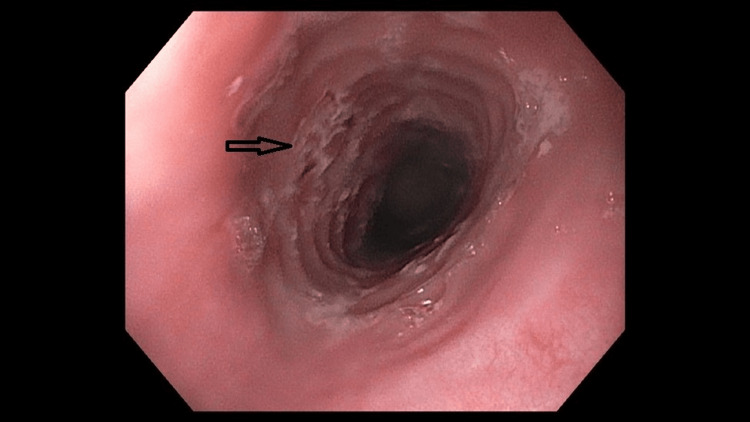
Herpes simplex esophagitis with ulcerations in the distal esophagus

**Figure 2:**
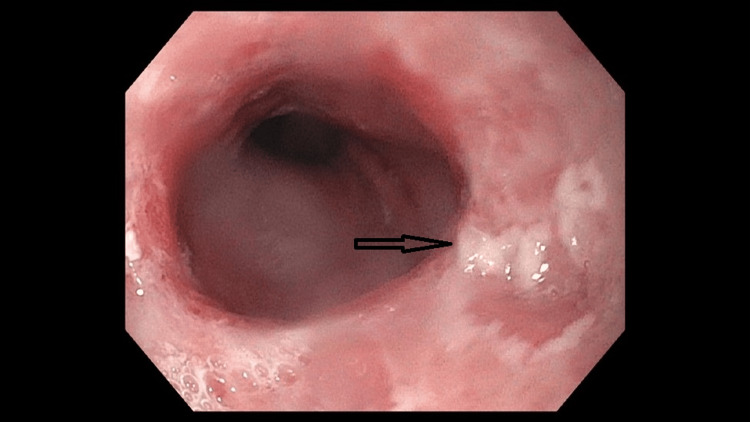
Severe esophagitis in the distal esophagus

**Figure 3:**
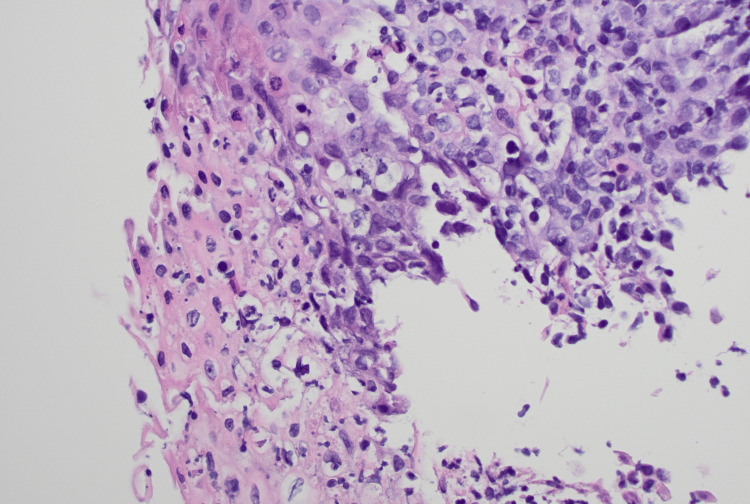
Focal areas of the ulcer bed show classic viral inclusions of the herpes simplex virus

**Figure 4:**
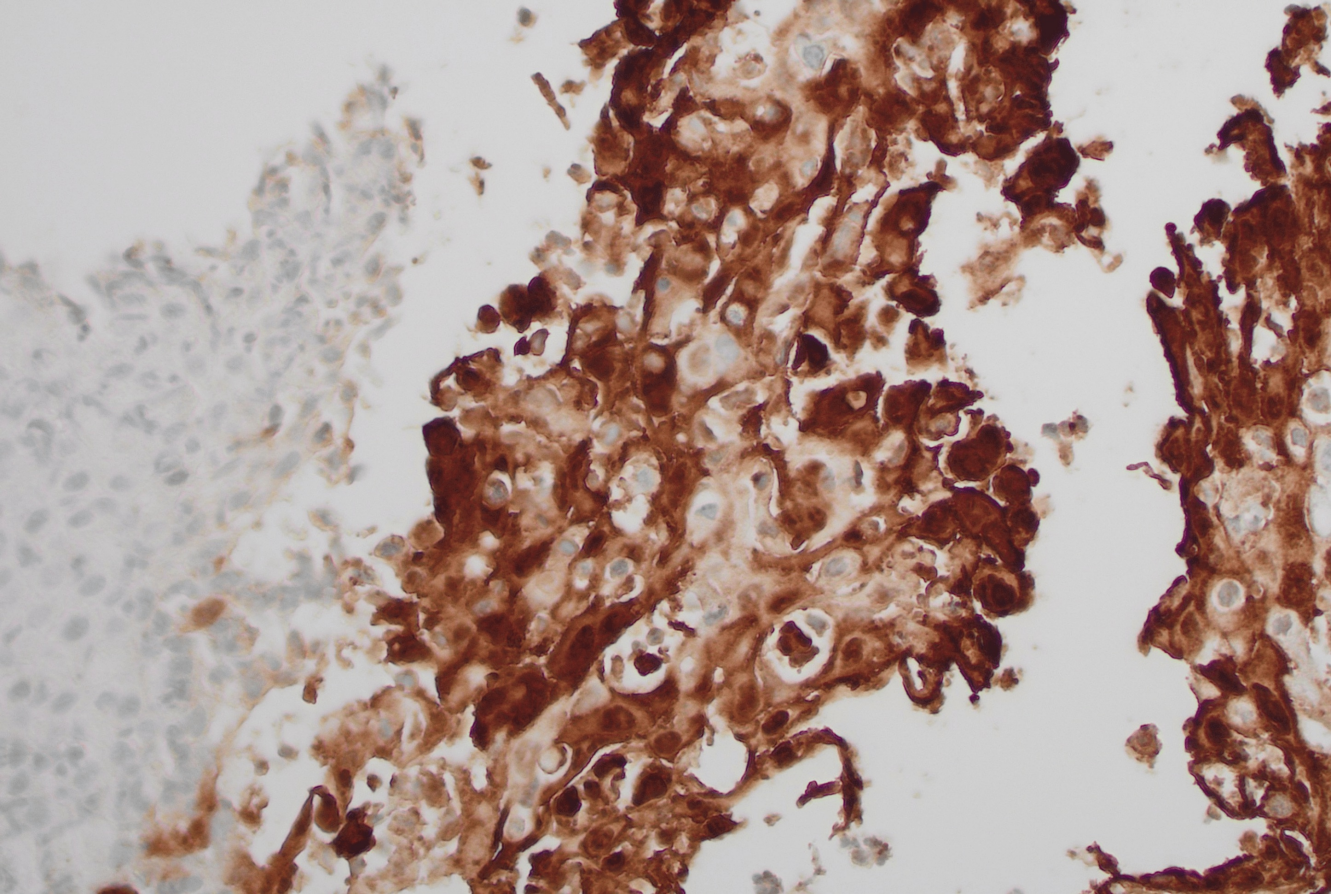
Herpes simplex virus immunohistochemical staining

On the third visit, gastroenterology was consulted, and an esophagogastroduodenoscopy (EGD) was performed, revealing severe esophagitis over a 10 cm segment (Figures 1, 2). Biopsies confirmed the presence of the herpes simplex virus with extensive ulceration, as shown by viral inclusions (Figure 3) and HSV immunohistochemical staining (Figure 4). Staining for cytomegalovirus (CMV) and fungi was negative.

Due to the severity of esophagitis and odynophagia, the patient was initially managed with intravenous (IV) acyclovir (5 mg/kg every 8 hours), which was later transitioned to oral acyclovir (400 mg three times daily) upon discharge from the hospital. The infectious disease team recommended a 14-day course of acyclovir. Viscous lidocaine and intravenous proton pump inhibitors (PPIs) were administered and transitioned to oral PPIs upon discharge.

The patient’s dietary intake was advanced from clear liquids to a regular diet as tolerated. The patient was discharged in stable condition with instructions for continued oral acyclovir therapy.

## Discussion

HSV esophagitis in an immunocompetent patient is rare, but it is clinically important and requires a high index of suspicion for the diagnosis. While it is commonly associated with immunocompromised states, this case shows the occurrence in a 64-year-old male without obvious immune compromise. It is possible that esophageal mucosal barrier disruption due to chronic GERD [2,7] and age-related decline in immunity [8] played a role. HSV esophagitis has been reported to have a strong male predominance of approximately 3:1 with a mean age of 35 [2,3]. Odynophagia is the most common symptom in 61-76% of cases, followed by fever (45-52%), retrosternal chest pain (46%), heartburn (50%), and dysphagia [2,3]. In about one quarter of cases, a prodrome of systemic or upper respiratory symptoms may precede esophageal symptoms [2]. The patient’s presentation with severe burning chest pain and difficulty swallowing initially suggested an exacerbation of reflux esophagitis. Nevertheless, the severity of symptoms despite proton pump inhibitor therapy necessitated additional investigation.

Histopathologic examination or viral culture of esophageal specimens is needed to make a diagnosis [4], and endoscopic biopsy was key in diagnosing HSV esophagitis in this case. Multiple ulcers are seen in the majority of cases (59-87%) on endoscopy, most commonly involving the distal esophagus (64%) [9]. Histopathology typically shows viral cytopathic changes such as multinucleated giant cells, ground-glass nuclei, and Cowdry type A inclusions. Tissue viral culture can improve diagnostic sensitivity, as can viral genome detection by PCR and immunohistochemistry [2]. Quantitative real-time PCR on esophageal biopsy specimens has demonstrated 94.7% sensitivity for HSV-1 esophagitis, with a viral threshold of 2.5 x 10⁴ copies achieving 100% specificity, providing a valuable adjunct when histopathologic findings are uncertain [10]. 

When standard treatments fail, alternative diagnoses should be considered, even in patients without classic risk factors for viral esophagitis. Antiviral therapy with acyclovir is the treatment of choice in managing HSV esophagitis [6]. The rapid symptomatic improvement observed after initiation of acyclovir is consistent with prior reports [2,11] and supports early intervention once the diagnosis is suspected or confirmed. Although HSV esophagitis in immunocompetent patients is generally considered self-limited [12], rare but life-threatening complications underscore the importance of prompt treatment. Spontaneous esophageal perforation due to HSV esophagitis has been described [13]. The potential for severe pain, dehydration, repeated presentations, as seen here, and rare complications such as esophageal perforation and upper GI bleeding [3,6] make a strong practical case for prompt therapy.

## Conclusions

This case highlights HSV esophagitis as an important yet uncommon diagnosis in immunocompetent patients presenting with severe or atypical esophageal symptoms. Although the patient lacked traditional risk factors for immune compromise, the combination of underlying GERD and a clinical course that did not respond to initial PPI therapy supported a broader differential and a more comprehensive diagnostic approach. Endoscopic evaluation with biopsy ultimately confirmed HSV esophagitis and guided management. Intravenous acyclovir followed by oral therapy led to rapid symptomatic improvement, underscoring the benefit of early recognition and timely antiviral treatment in preventing prolonged illness and complications. Ongoing awareness and reporting of similar cases may help clinicians recognize this entity sooner and manage it more effectively.
